# Thoracic aortic aneurysms exerting high extrinsic pressure on the airway

**DOI:** 10.1186/s13019-019-0992-x

**Published:** 2019-09-18

**Authors:** Hanna Jung, Young Woo Do, Sang Yub Lee, Youngok Lee, Tak Hyuk Oh, Gun Jik Kim

**Affiliations:** 1 Department of Thoracic and Cardiovascular Surgery, School of Medicine, Kyungpook National University, Kyungpook National University Hospital, 130 Dongdeok-ro, Jung-gu, Daegu, 41944 Republic of Korea; 2 Department of Radiology, School of Medicine, Kyungpook National University, Kyungpook National University Hospital, Daegu, 41944 Republic of Korea

**Keywords:** Airway obstruction, Thoracic aortic aneurysm, Stents

## Abstract

**Background:**

Thoracic aortic aneurysms, although mostly asymptomatic, are life threatening owing to the risk of rupture. Moreover, the extrinsic pressure of a ruptured aneurysm may encroach the mediastinum.

**Case presentation:**

A 74-year-old woman diagnosed with ruptured descending thoracic aortic aneurysm compressing the lower trachea and both main bronchi underwent thoracic endovascular aortic repair; however, the extrinsic pressure on the airway persisted. Following failing of endobronchial silicon stents insertion, extracorporeal membrane oxygenation support was required, and endobronchial metallic stents were inserted. The patients’ hypoventilation resolved, and the patient was withdrawn from the ventilator.

**Conclusions:**

Technological improvement in endovascular or endobronchial procedures has provided more options for managing complex cases. However, we must be aware of how high the extrinsic pressure might be before management and take steps to minimize complications.

## Background

Thoracic aortic aneurysms are commonly undiagnosed until the appearance of symptoms, and the patient might be fortunate if the symptoms related to aneurysmal enlargement appear and are diagnosed before rupture [1]. However, diagnosis at the time of rupture may be life threatening. Moreover, extrinsic pressure of the ruptured aneurysm may also encroach the organs in the mediastinum.

Recently, the use of endovascular stent grafts has increased owing to development of procedures that have lower risk and are lesser invasive and lesser traumatic than surgical operations for the treatment of thoracic aortic aneurysm [2]. However, in complicated ruptured cases, the extrinsic pressure of the aneurysm may not be relieved with the stent graft, resulting in the need of an additional procedure or a period time for aorta remodeling.

Here, we report our experience in managing extrinsic pressure on the airway related to the rupture of the descending thoracic aortic aneurysm.

## Case presentation

**Fig. 1:**
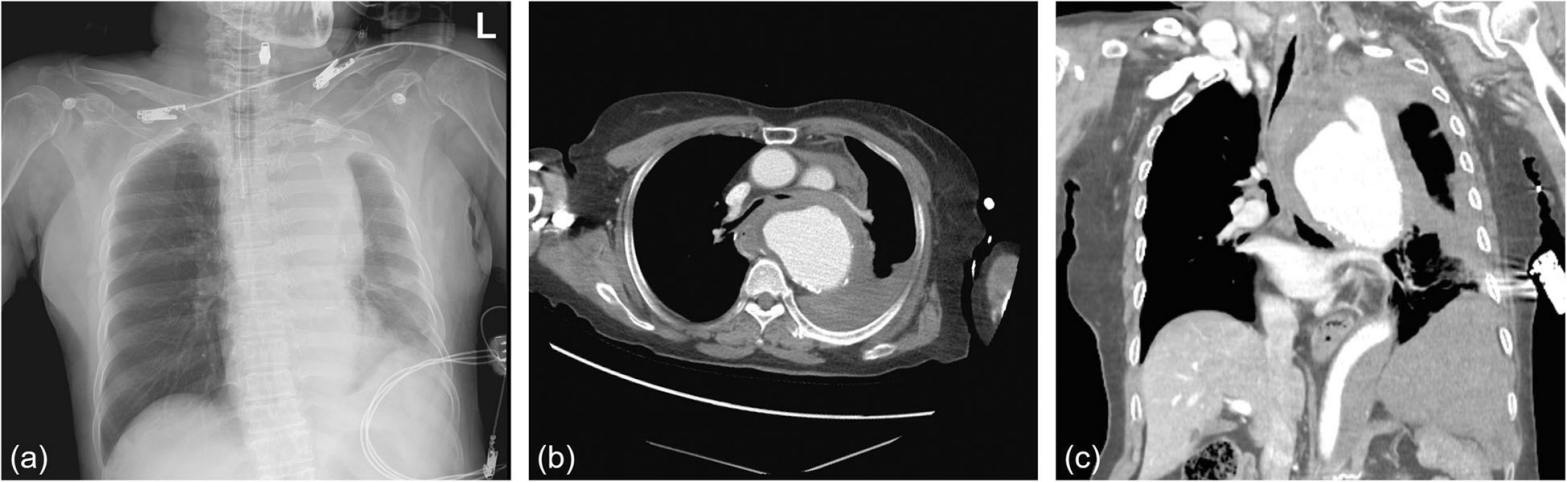
(**a**) Chest radiography and (**b**, **c**) computed tomography of the chest after the aneurysmal rupture. The maximal diameter of the aneurysm is 9 cm at the level of the carina

A 74-year-old woman with sudden chest pain and dyspnea was admitted to the emergency room and intubated on arrival owing to disturbed consciousness. She had hypertension but no history of asthma or chronic obstructive pulmonary disease. Chest radiography showed mediastinal widening (Fig. 1a). Computed tomography (CT) of the chest revealed rupture of the descending thoracic aortic aneurysm, which compressed the lower trachea and both main bronchi. The maximal diameter of the aneurysm at the level of the carina was 9 cm (Fig. 1b, c).

Even with aneurysmal rupture, after intubation and during the diagnosis, her vital signs were maintained stable enough to be transferred to our hospital. We performed an emergency thoracic endovascular aortic repair (TEVAR, seal stent graft S&G Biotech Inc., Yongin-si, Korea, proximal 34 × 30 × 110 and distal 28 × 110). TEVAR was performed uneventfully and following admission to the intensive care unit, her vital signs were stable.

**Fig. 2:**
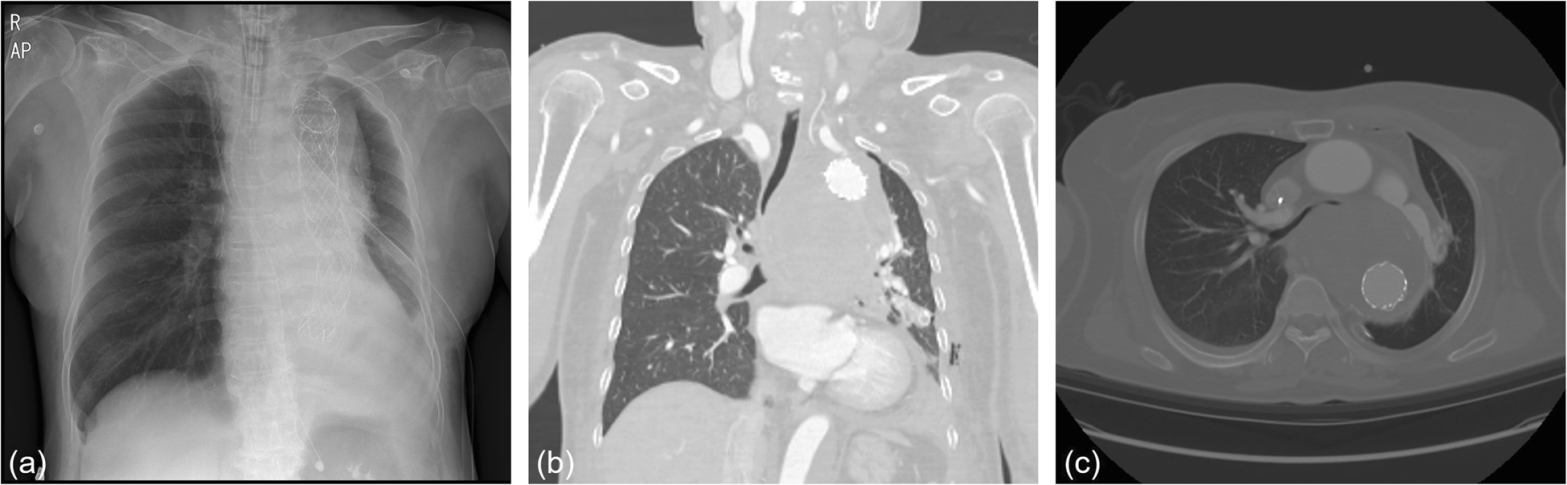
(**a**) Chest radiography and (**b**, **c**) computed tomography of the chest after thoracic endovascular aortic repair (TEVAR, proximal 34 × 30 × 110 and distal 28 × 110). The hematoma of the aneurysm still compressing the lower trachea and both main bronchi

However, even with the mechanical ventilator support, carbon dioxide (CO_2_) retention was not easily resolved, and sudden oxygen desaturation with unusually high airway pressure and low tidal volume was repeated. Moreover, the suction tube was difficult or unable to pass the endotracheal tube. We were aware that the patient had airway compression in the initial CT, but anticipated that the extrinsic compression pressure of the ruptured aneurysm would be relieved after the TEVAR. Due to the failure to wean the patient from the mechanical ventilator, the follow-up chest CT was performed. Despite the stent graft in the aorta (Fig. 2a), the hematoma of the ruptured aneurysm continued compressing the lower trachea and both main bronchi (Fig. 2b, c).

We planned to insert a silicone bronchial stent under rigid bronchoscopy. However, after removal of the endotracheal tube, the rigid bronchoscopy was unable to enter the trachea. Further, re-intubation was not possible even with the anesthesiologist owing to the extrinsic pressure on the airway. Because the patient crashed owing to respiratory failure, she immediately underwent veno-veno extracorporeal membrane oxygenation (VV ECMO) support and subsequently, insertion of the silicone stent. However, the day after insertion, the silicone stent in the bronchi migrated upward owing to the extrinsic pressure and obstructed the carina. Therefore it had to removed.

**Fig. 3:**
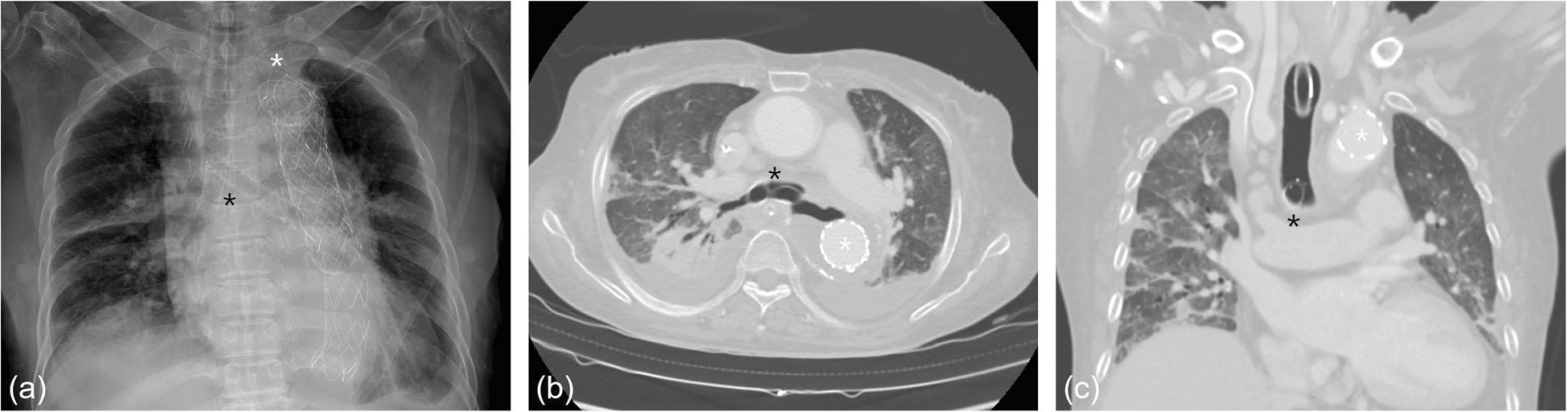
(**a**) Endobronchial stents (Nitinol Stent, right 10 × 40 mm and left 10 × 60 mm) are inserted in the both main bronchi. (**b**, **c**) Computed tomography of the chest after TEVAR (white start) and endobronchial stenting (black star)

Through multidisciplinary cooperation with radiology, we planned to insert a metallic stent. Under wire-guided fluoroscopy, a nitinol metallic stent (Cordis S.M.A.R.T.® CONTROL™ Self-Expanding Nitinol Stent, Florida, United States, right 10 × 40 mm and left 10 × 60 mm) was inserted into both main bronchi (Fig. 3a). The day after nitinol stent insertion, hypoventilation and CO_2_ retention improved, and the patient was able to remove VV ECMO. During the several invasive procedures, the patient had laceration of the trachea and pneumothorax, but eventually she could tolerate withdrawal of ventilator support with widened trachea and main bronchi (Fig. 3b, c) and was transferred to local hospital.

## Discussion and conclusions

DeBakey et al. reported an 8% incidence of airway compromise associated with aortic aneurysm without differentiating between the types of aneurysms [3]. Tracheobronchial compression resulting in stridor, wheezing, cough, or pneumonitis is well-known but uncommon complication of aneurysms of the aortic arch and a rare complication of descending thoracic aortic aneurysm [4]. Descending thoracic aortic aneurysm rupture, despite emergency TEVAR could progress into complex case by compression of the adjacent thoracic structures in the mediastinum. As in our case, TEVAR was not adequate to resolve all the patient’s symptoms, thus requiring endobronchial stenting in addition.

Although the management of the case to this point was based on our knowledge about the condition, we had not accounted for the extrinsic pressure of the ruptured aneurysm. The chest CT revealed airway compression, but we did not consider it seriously. On retrospection, low tidal volume, CO_2_ retention, and failure of insertion of the suction tube were clues indicating us to exercise caution. With airway compression of the lower trachea and both main bronchi, the patient was not indicated for emergency tracheostomy or cricothyroidotomy. Although the patient’s vital signs were stable prior to rigid bronchoscopy, VV ECMO should have prepared for respiratory failure. Had we considered the extrinsic pressure on the airway, we might have considered inserting the metallic stent in the bronchi the first time.

There are reports about multistep management of thoracic aortic aneurysm using endovascular and endobronchial approach [1, 2, 5, 6] or VV ECMO application during variant airway problems [7, 8]. However, none of them reported about the extrinsic pressure of the ruptured aneurysm and how high it might be.

Fortunately, our patient did not develop hypoxic brain damage during the several hypoxic event or cardiopulmonary resuscitations. It would have been better if the patient had not gone through such events that could have prevented.

TEVAR has been widely used in the recent years as a minimally invasive treatment modality. However, TEVAR may not always immediately resolve symptoms associated with airway compression. Endobronchial procedures may have been developed over the past decade but their application has increased for treatment of airway stenosis caused by extrinsic compression [2]. Technological improvement has given us more options for the management of complex cases. However, we must adopt measure to minimizes unnecessary events or complications during management of these patients.

## Data Availability

Not applicable.

## Abbreviations

CO_2_: Carbon dioxide
CT: Computed tomography
TEVAR: Thoracic endovascular aortic repair
VV ECMO: Veno-veno extracorporeal membrane oxygenation
